# rTMS combined with CBT as a next step in antidepressant non-responders: a study protocol for a randomized comparison with current antidepressant treatment approaches

**DOI:** 10.1186/s12888-022-03732-6

**Published:** 2022-02-05

**Authors:** Iris Dalhuisen, Filip Smit, Jan Spijker, Iris van Oostrom, Eric van Exel, Hans van Mierlo, Dieuwertje de Waardt, Martijn Arns, Indira Tendolkar, Philip van Eijndhoven

**Affiliations:** 1 Department of Psychiatry, Radboud University Medical Center, Nijmegen, The Netherlands; 2 Donders Institute for Brain Cognition and Behavior, Centre for Medical Neuroscience, Nijmegen, The Netherlands; 3 Department of Clinical Psychology and Department of Epidemiology and Biostatistics, Amsterdam University Medical Centers, location VUmc, Amsterdam, The Netherlands; 4 Department of Mental Health and Prevention, Trimbos Institute - Netherlands Institute of Mental Health and Addiction, Utrecht, The Netherlands; 5 Depression Expertise Centre, Pro Persona Mental Health Care, Nijmegen, The Netherlands; 6 Behavioral Science Institute, Radboud University, Nijmegen, The Netherlands; 7 Neurocare, Nijmegen, The Netherlands; 8 Department of Psychiatry, GGZ inGeest Specialized Mental Health Care, Amsterdam, Netherlands; 9 Department of Psychiatry & Psychology, St. Antonius Hospital, Utrecht/Nieuwegein, The Netherlands; 10 Department of Psychiatry, ETZ Hospital (Elisabeth-TweeSteden Ziekenhuis), Tilburg, The Netherlands; 11 Research Institute Brainclinics, Nijmegen, The Netherlands; 12 Faculty of Psychology and Neuroscience, Maastricht University, Maastricht, the Netherlands

**Keywords:** rTMS, Depression, RCT, (cost)-effectiveness

## Abstract

**Background:**

Major depressive disorder (MDD) is one of the most common psychiatric disorders, however, current treatment options are insufficiently effective for about 35% of patients, resulting in treatment-resistant depression (TRD). Repetitive transcranial magnetic stimulation (rTMS) is a form of non-invasive neuromodulation that is effective in treating TRD. Not much is known about the comparative efficacy of rTMS and other treatments and their timing within the treatment algorithm, making it difficult for the treating physician to establish when rTMS is best offered as a treatment option. This study aims to investigate the (cost-)effectiveness of rTMS (in combination with cognitive behavioral therapy (CBT) and continued antidepressant medication), compared to the next step in the treatment algorithm. This will be done in a sample of patients with treatment resistant non-psychotic unipolar depression.

**Methods:**

In this pragmatic multicenter randomized controlled trial 132 patients with MDD are randomized to either rTMS or the next pharmacological step within the current treatment protocol (a switch to a tricyclic antidepressant or augmentation with lithium or a second-generation antipsychotic). Both groups also receive CBT. The trial consists of 8 weeks of unblinded treatment followed by follow-up of the cohort at four and 6 months. A subgroup of patients (*n* = 92) will have an extended follow-up at nine and 12 months to assess effect decay or retention. We expect that rTMS is more (cost-)effective than medication in reducing depressive symptoms in patients with TRD. We will also explore the effects of both treatments on symptoms associated with depression, e.g. anhedonia and rumination, as well as the effect of expectations regarding the treatments on its effectiveness.

**Discussion:**

The present trial aims to inform clinical decision making about whether rTMS should be considered as a treatment option in patients with TRD. The results may improve treatment outcomes in patients with TRD and may facilitate adoption of rTMS in the treatment algorithm for depression and its implementation in clinical practice.

**Trial registration:**

This trial is registered within the Netherlands Trial Register (code: NL7628, date: March 29th 2019).

## Background

Major depressive disorder (MDD) is one of the most common psychiatric disorders, with over three hundred million people affected worldwide [1]. The mortality rate is nearly twice as high in individuals with MDD, not only in clinical but even in subclinical depression, and MDD is the second leading cause of years lived with disability (YLD), with an estimated global loss of more than 63 million healthy years in 2010 [2, 3]. Next to the burden for patients and those who care for them, MDD has a substantial economic impact, with estimated yearly costs of €91 billion and $236 billion in Europe and the U.S.A., respectively [4, 5]. Although antidepressants and psychotherapy (e.g. cognitive behavioral therapy, CBT) offer effective treatment options for MDD, a substantial group of patients does not respond adequately to treatment. Specifically, the percentage of patients that achieves remission after an antidepressant trial drops dramatically from approximately 35% for the first two trials to only 13% for a third or fourth trial, whereas the number of side effects and patients that stop treatment increases substantially [6]. Up to 35% of patients fail to respond to first line treatments and suffer from treatment-resistant depression (TRD) [6]. However, there is no unified definition of TRD (sometimes also referred to as difficult-to-treat-depression; DTD), which has been operationalized in various ways [7, 8]. For example, the Thase and Rush staging method defines five levels of treatment resistance, based on the number and classes of failed antidepressant treatment trials [9]. Alternately, Conway and colleagues propose a heuristic two stage-model of TRD with moderate and severe treatment resistance, largely based on the inflection point that is seen after two antidepressant treatment trials [10]. At this point, a different treatment modality with a different mechanism of action may be preferred over yet another antidepressant medication trial. In severe cases electroconvulsive therapy (ECT) would be such an option. However, despite its superior effectiveness, ECT is also invasive and comes with a risk of substantial side effects such as short term memory loss. Ideally, a less invasive treatment option would be available when antidepressants fail. Furthermore, irrespective of staging as a function of the number of failed treatments, longer periods of insufficient treatment lead to a higher risk of chronicity, comorbidity and suicidality, emphasizing the need for effective treatment options for patients with TRD [11].

Repetitive transcranial magnetic stimulation (rTMS) is a non-invasive neurostimulation method that is increasingly being used to treat MDD, with promising effects in TRD [12]. The therapeutic effect of rTMS is achieved by delivering magnetic pulses through a coil that is positioned above the head. The magnetic field induces an electrical current in the underlying cortex that modulates neuronal activity. Based on the observation of dysfunctional dorsolateral prefrontal cortex (DLPFC) activity in depressed patients in neuroimaging studies, the DLPFC was selected as the primary target for MDD [13, 14]. High-frequency stimulation over the left DLPFC or low-frequency stimulation over the right DLPFC can both be applied in order to modulate the brain circuits that are involved in depression [15]. In recent years a sufficient body of evidence has been build up to establish the definite antidepressant efficacy of high frequent rTMS over the left DLPFC and probable antidepressant efficacy of low frequent rTMS over the right DLPFC [16]. A recent meta-analysis that included 57 RCTs showed a significantly larger depressive symptom reduction after active rTMS compared to sham (Hedges’ g = − 0.832), and similar results were found for response and remission rate [17]. Evidence from a large naturalistic study (*n* = 196) in one of the participating centers indicates that combining rTMS with CBT even yields additional effects, contributing to robust remission rates of 56% in TRD [18]. This additive effect of combining rTMS with CBT has also been established in other psychiatric disorders, such as post-traumatic stress disorder and obsessive-compulsive disorder, as well as for combining antidepressant medication with CBT [19–22].

Despite being approved as a treatment for patients with TRD, the exact position of rTMS in the treatment algorithms of depression remains unclear. The biological steps within these algorithms usually follow a stepped-care approach, starting with multiple, subsequent psychopharmacological steps and ECT as the last step. Not much is known about the comparative efficacy of rTMS to other biological steps, although studies have compared rTMS to venlafaxine and ECT [23, 24]. Importantly, the efficacy of these biological treatments may also depend on the level of treatment resistance. It is therefore difficult for the treating physician to decide when rTMS should be considered as a treatment option for his or her patient. A few studies have performed economic evaluations, comparing rTMS to other antidepressant treatments. Results on the cost-effectiveness of rTMS compared to ECT show mixed results, with some studies suggesting rTMS and others ECT as the most cost-effective treatment [25–28], where the level of treatment resistance and severity of depression may be the crucial factor. When compared to antidepressant medication, rTMS resulted in slightly more quality of life at lower costs [29, 30]. However, no studies have assessed the cost-effectiveness of rTMS as a follow-up step in patients with moderate level of treatment resistance.

Therefore, we want to examine the comparative efficacy of rTMS combined with CBT as an alternative to the current next pharmacological steps (i.e., switch or augmentation of antidepressant) in a randomized controlled trial. We propose to investigate the effectiveness of rTMS in combination with CBT and continued antidepressant medication compared to the next step in the treatment algorithm, in a sample of patients with non-psychotic unipolar depression, who did not respond to two adequate antidepressant treatments. We aim at testing the effectiveness of both treatments in terms of clinical improvement and the prevention of recurrence over the course of 6 months, as well as the cost-effectiveness. Furthermore, we will examine effect decay or preservation up until 12 months. We also aim to explore the effects of the two treatments on specific symptoms associated with depression, such as anhedonia, anxiety, rumination, cognitive reactivity, and sleep. Finally, we aim to assess the effect of expectations regarding the treatment on its effectiveness.

## Methods

### Study design

**Fig. 1:**
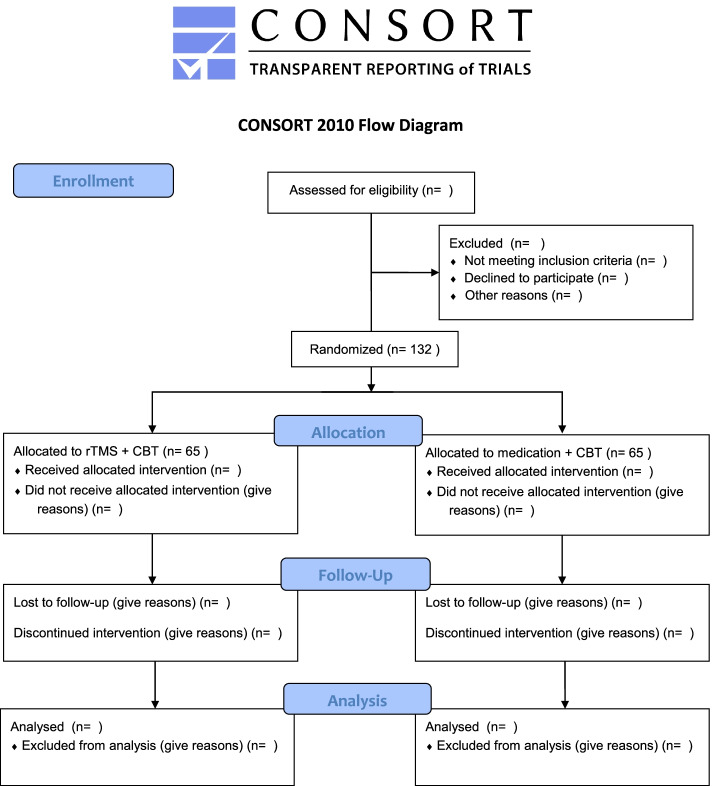
CONSORT flowchart

In this pragmatic randomized controlled trial (RCT), patients are randomly assigned to one of two treatment conditions. In the first condition, treatment consists of rTMS combined with CBT and continued antidepressant medication, which will hereafter be referred to as the rTMS arm. In the second condition, treatment consists of a switch from the current antidepressant medication to a tricyclic antidepressant (TCA) or augmentation with lithium or a second-generation antipsychotic, also in combination with CBT. This will be referred to as the medication arm. The trial consists of 8 weeks of unblinded treatment followed by a follow-up of the cohort at four and 6 months from baseline onward. For a subgroup of the patients, an extended follow-up will take place at nine and 12 months from baseline onward to assess effect decay or preservation. See Fig. 1 for the CONSORT flowchart. Patients in the medication arm who did not recover to the medication intervention can receive rTMS after completion of the first 8 weeks of treatment.

### Aims and hypotheses

Our primary objective is to quantify whether 8 weeks of treatment with rTMS will outperform medication in terms of improvement of depressive symptoms. We expect that rTMS is more effective than medication in reducing depressive symptoms in patients with TRD.

Our secondary objective is to perform a health evaluation consisting of a cost-effectiveness analysis (CEA) with treatment response and remission as outcomes and a cost-utility analysis (CUA) with quality adjusted life years (QALYs) gained as outcome. The CEA and CUA will be performed along-side the trial. We expect rTMS to be more cost-effective than medication.

Finally, we aim to perform exploratory analyses of the effect of rTMS on other symptoms associated with depression, compared to the effect of new medication on these symptoms. The symptoms consist of suicidality, anhedonia, anxiety, rumination, cognitive reactivity, and sleep. A second exploratory objective is to assess the effect of expectations regarding the treatment on its effectiveness.

### Participants

We aim to include 132 adult patients diagnosed with moderate to severe (HDRS > 16) unipolar MDD without psychotic symptoms, with failed response to at least two treatment trials with adequately dosed antidepressants and a current episode of less than 2 years. Exclusion criteria are: lifetime diagnosis of bipolar disorder, schizophrenia or schizoaffective disorder; current diagnosis of substance abuse disorder or organic brain syndrome; the presence of a concurrent significant medical condition impeding the ability to participate; previous treatment with rTMS or ECT; epilepsy, convulsion or seizure; serious head trauma or brain surgery; large or ferromagnetic metal parts in the head (except for a dental wire); implanted cardiac pacemaker or neurostimulator; or pregnancy. A subset of 92 patients will be part of the extended follow-up up to 12 months.

### Sample size calculation

The sample size calculation is based on the outcome of the rTMS arm in comparison to the medication arm. To flag up a clinically significant difference between rTMS and medication, we took an effect size of d = 0.33, which corresponds to the lower boundary of a medium clinically meaningful effect [31] and is similar to effect sizes for research on antidepressant medication [32]. Alpha will be set at 0.05, with a power (1-beta) of 0.80. We will employ a two-sided repeated measures ANCOVA, with a total of six repeated measurements (baseline and measurements corresponding to rTMS treatment 5, 10, 15, 20, and 25). See also Table 1 for an overview and timepoints of the measurements. Based on HDRS-17 data from one of our previous studies, we expect the correlation between baseline and follow-up to be *r* = 0.59, and the correlation between follow-up measurements to be *r* = 0.69. The sample size calculation was performed in STATA version 15.1 and resulted in an estimated required sample size of 59 participants per condition. Compensating for possibly 10% loss to follow-up, the total sample size at baseline will be increased to 132 participants.

**Table 1:** Overview of rTMS sessions and measurements during the treatment period. For participants in the medication arm, measurements will be done on days corresponding to treatment day 5, 10, 15, 20, and 25 of rTMS treatment

	Week 1					Week 2					Week 3					Week 4				
Day	1	2	3	4	5	1	2	3	4	5	1	2	3	4	5	1	2	3	4	5
rTMS	x	x		x	x	x	x		x	x	x	x		x	x	x		x		x
HDRS						x						x								x
BDI-II						x						x								x
	Week 5					Week 6					Week 7					Week 8				
Day	1	2	3	4	5	1	2	3	4	5	1	2	3	4	5	1	2	3	4	5
rTMS	x		x		x	x		x		x		x		x			x		x	
HDRS								x											x	
BDI-II								x											x	

### Procedure

This is a multicenter trial, taking place within the Dutch specialized mental health care setting. Patients are recruited and treated at Radboud University Medical Center (Radboudumc; Nijmegen), Pro Persona mental health care (Nijmegen, Arnhem), neurocare (Nijmegen, Arnhem, Eindhoven, Den Haag), the Elisabeth TweeSteden Hospital (ETZ; Tilburg), GGZ inGeest mental health care (Amsterdam), the St. Antonius Hospital (Utrecht, Nieuwegein), and also recruited via the Dutch Depression Foundation. If patients are eligible for participation, the psychiatrists or psychologists approach them to participate in the trial, after which they are contacted by a researcher. Study information is provided and patients are contacted again after at least 24 h to discuss participation and discuss any remaining questions. If patients decide to participate, they are invited for a research intake, where they are asked to sign the informed consent form and all baseline measurements are conducted. Afterwards, patients are randomized by the principal investigator (see below).

At baseline and during treatment, questionnaires will be administered by the clinician or researcher. Patients also fill in questionnaires themselves, which are sent via email using the secure online data capture program for clinical trials, Castor EDC [33]. The questionnaires can be filled out via any personal device (e.g. computer, tablet, smartphone). The expected study duration is 39 months, and inclusion of patients started in August 2019.

### Ethics

The present study was approved by the Medical Ethics Committee of Arnhem-Nijmegen (NL68540.091.19) and is registered within the Netherlands Trial Registry (NL7628). All participants will give written informed consent according to the Declaration of Helsinki before entering the study and will be explicitly informed that they can withdraw from participation at any time without any further explanation. Participants will be given an identification code, which will be used for further data acquisition. Participant names with their identification codes will be kept securely and only accessible by the principal investigator and coordinating researcher. A trained and independent monitor will be assigned to monitor the research process, which includes monitoring the completeness of informed consent, data safety, and correspondence with the ethics committee. Although these are not expected to occur, serious adverse events will be reported to the Medical Ethics Committee.

### Randomization

Participants will be randomly assigned to either the rTMS or medication arm. Randomization will be done via Castor EDC and will be stratified for treatment center, total number of depressive episodes, and current depression severity (moderate: HDRS-17 score of 17–23; severe: HDRS-17 score of > 23). Castor EDC randomizes participants with a four and six block design. More information about the randomization algorithm can be found on Castor’s website (https://helpdesk.castoredc.com/article/50-the-randomizationalgorithm-in-castor).

### Interventions

#### rTMS

rTMS treatment will be performed using a Magstim Rapid 2 (Radboudumc) or a Deymed DuoMag XT-35 or XT-100 equipment (neurocare and ETZ) using generic figure-8 coils). All rTMS parameters used in the proposed study are within the range considered safe according to the latest published safety guidelines [34, 35]. Firstly, the resting motor threshold (rMT) will be defined in each participant as the minimal stimulation intensity evoking an MEP of ≥0.05 mV in 50% of the trials in the muscle of the right thumb (M. abductor pollicis brevis). Note that rMT will be determined at the beginning of every week before the first treatment session. We will use BeamF3 (freely available software at (http://clinicalresearcher.org/F3/calculate.php) to find the DLPFC, which is based on electrode position F3 from the EEG 10–20 system [36]. A detailed description of this method can be found in Beam et al., (2009) [36]. The coil will be positioned over this location at an angle of 45° relative to the midline.

rTMS will be conducted using a high-frequent protocol, applying 60 trains of 10 Hz pulses with a duration of 5 s and an inter-train interval of 25 s over the left DLPFC (50 pulses per train, 3000 pulses per session), with an intensity of 120% of the rMT. Twenty-five rTMS sessions will be given over the course of 8 weeks, following a schema of 4, 4, 4, 3, 3, 3, 2, 2 sessions per week. After a minimum of fifteen sessions (corresponding to 4 weeks), treatment may be ended. This is only possible when the scores on the HDRS-17 decrease to a minimum of symptoms (score < 8, in remission) for at least two measurements in a row. However, when the scores still show a decline, the patient is still improving and will likely progress even further. The rTMS sessions should be continued until the patient’s HDRS scores remain below 8 for two measurements in a row but no longer than 8 weeks (given the set-up of the approved ZonMW grant). Table 1 shows an overview of the rTMS treatments and measurements during the eight-week treatment period. Patients responding to rTMS are allowed to receive booster sessions in case of relapse.

#### CBT

All patients will receive sessions of cognitive behavioral therapy, either in a group or individually, and at least once a week. This may differ between patients, as it would during usual care. Information on the form and frequency of CBT will be recorded in Castor EDC.

#### Medication

Treatment as usual will consist of protocolled pharmacological treatment steps that are prescribed within the medication algorithm of the Dutch guideline. This will consist of either a switch from the current antidepressant medication to a tricyclic antidepressant (TCA) or augmentation of the current antidepressant medication with lithium or a second generation antipsychotic [37]. Information on the type, dose and duration of medication will be recorded in Castor EDC.

#### Measures

**Fig. 2:**
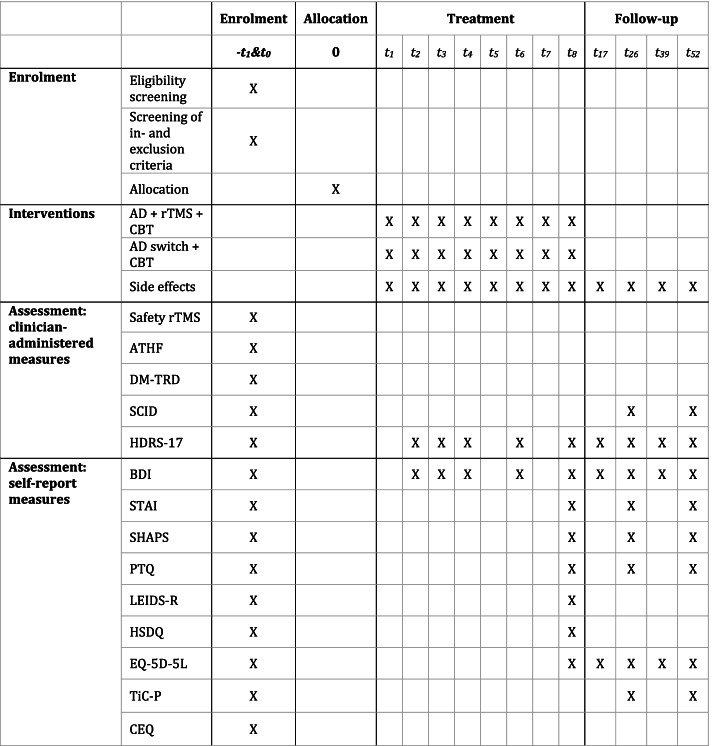
Assessment schedule

See Fig. 2 for a schedule of the assessments.

### Primary objective - depressive symptoms

Section A, B and D of the Structured Clinical Interview for DSM 5 disorders (SCID-I; 2018) will be used at baseline and after six and 12 months to assess depressive episodes and possible relapses/recurrences at follow up and extended follow-up. During intake, the SCID will also be used to assess presence of bipolar disorder or psychotic features. The SCID takes about 20 min to complete.

The Hamilton Depression Rating Scale 17 item version is an observer rated scale used to assess the severity of depressive symptoms (HDRS-17) [38]. The HDRS-17 is assessed during screening, treatment, follow-up and extended follow-up. Completion takes approximately 15 min. During the eight-week treatment period, the HDRS-17 will be administered every five rTMS treatments, and on corresponding days for participants in the medication arm. See Fig. 2 for the schedule during treatment. Response is defined as a ≥ 50% reduction in score on the HDRS-17, whereas remission is defined as a score of < 8. Response and remission will be determined after 8 weeks of treatment and at the final follow-up assessment of the RCT at 6 months after baseline.

The Beck Depression Inventory will be used in order to measure individual differences in depressive symptoms (BDI-II) [39]. Participants will be asked to fill in the BDI-II once every five rTMS sessions or on corresponding days when they are in the medication arm. See Fig. 2 for the schedule during treatment. Participants have to indicate on 21 items in how far these items describe themselves on a scale ranging from 0 to 3 resulting in a sum score between 0 and 63. The total score can further be divided into four ranges, describing the severity of depressive symptoms from minimal (0–13), mild (14-19), moderate (20-28) to serious depressive symptoms (29–63). Completion takes about 5 min.

Two questionnaires will be used to assess and quantify the level of treatment resistance. The Dutch Measure for quantification of Treatment Resistance in Depression (DM-TRD) quantifies treatment-resistant depression by assessing treatments in the depressive episode as well as functional impairment, comorbid anxiety, personality disorders and psychosocial stressors [40]. The DM-TRD generally takes 10-20 min to complete after the diagnostic interview. The Antidepressant Treatment History Form (ATHF) can be administered in 10 min or less, depending on the number of antidepressant trials of a patient [41].

### Secondary objective - health-related quality of life and healthcare costs

To measure (changes in) health-related quality of life of the patients the EuroQol 5-dimensions 5-levels (EQ-5D-5L) will be used. The EQ-5D-5L describes health states over five domains (mobility, self-care, usual activities, pain/discomfort and anxiety/depression) and can be completed in less than 5 min. Dutch tariffs will be applied to each of the health states to obtain utilities, which are anchored at 0 (death) and 1 (full health). EQ-5D-5L utilities will be combined over time using the area under the curve (AUC) method to capture the cumulative QALY health gains over the entire six- or twelve-month follow-up period.

The Trimbos Institute and iMTA Cost questionnaire for Psychiatry (TiC-P) will be used for measuring health care usage (and the costs thereof), patients’ and their family’s out-of-pocket costs, and productivity losses owing to absenteeism and presenteeism. In the Netherlands the TiC-P is the most frequently used health care receipt questionnaire for health-economic evaluation. Completion of the TiC-P will take roughly 10 min.

### Exploratory analyses – specific symptoms associated with depression

The State/Trait Anxiety Inventory (STAI) is a self-report measure which has been proven reliable and sensitive in the assessment of both state and trait levels of anxiety. It is a standard international measure in anxiety research and its Dutch translation has been validated. Filling in the STAI takes approximately 5 min. The Snaith-Hamilton Pleasure Scale (SHAPS) measures anhedonia and takes 5 min to complete. To assess rumination and repetitive negative thinking, the Perseverative Thinking Questionnaire (PTQ) will be administered. The Dutch version shows satisfactory psychometric properties and can be completed in about 5 min. The Holland Sleep Disorders Questionnaire (HSDQ) is a self-assessment questionnaire for sleep disorders which can distinguish between the six sleep disorders classified in the International Classification of Sleep Disorders. The HSDQ can be completed in 10 min. The Leiden Index of Depression Sensitivity – Revised (LEIDS-R) measures cognitive reactivity and can be used as a marker of vulnerability to depression. It takes about 10 min to complete.

### Exploratory analyses – expectancy

The Credibility/Expectancy Questionnaire (CEQ) aims to assess the expectations patients have of a treatment. We have tailored this questionnaire to our study design. The questionnaire consists of eight questions, four of which concern the rTMS treatment, and four of which are about the medication treatment. Questions will be answered with a visual analog scale. Baseline expectations regarding antidepressant treatment have been shown to correlate with treatment outcome [42].

#### Analysis

The clinical outcome data will be analyzed and reported according to the CONSORT guidelines, i.e. primarily on intention-to-treat (ITT) basis.

### Description of the sample

Participant characteristics will be reported in Table 1 of the research paper, showing mean and standard deviation or percentage for each variable.

### Change in depression severity

Our primary outcome during the intervention is overall change in HDRS-17 depressive symptom severity during 8 weeks of treatment by condition (rTMS versus medication). Data will be collected at 6 timepoints: at baseline, and after 5, 10, 15, 20, and 25 rTMS treatment sessions (or corresponding days for participants in the medication arm). We will employ linear mixed modelling using marginal means, with HDRS-17 score as our dependent variable and treatment (rTMS or medication) as independent variable. Age and gender will be added as covariates, since these have been shown to affect the phenomenology of depression.

### Evaluation of treatment response and remission

One of our secondary outcomes is treatment response (50% reduction in HDRS-17 score) and remission (≤7 on HDRS-17) at 8 weeks, and 4 and 6 months. For a subgroup of patients, this data will also be available for 9 and 12 months, as part of the extended follow-up. For each treatment condition, the percentage of participants that responds or is in remission will be calculated, for each time point. Kaplan Meier survival estimates will be calculated based on outcome after the eight-week treatment period and during follow-up. Scores will be compared using Cox regression analysis to take into account both the rate of response or remission and the time needed to reach response or remission.

### Cost-effectiveness analysis

#### Costs

Three types of costs will be included: (1) healthcare costs including intervention costs (i.e. rTMS, pharmacotherapy, CBT sessions), (2) patients’ and their family’s costs for travel and informal care, and (3) costs stemming from productivity losses due to absenteeism and lesser efficiency while at work, both in paid work and volunteer jobs. Data on resource use (health care uptake, informal care, travel distances to health services, and productivity losses) will be collected with the latest version of the Trimbos/iMTA Questionnaire on Costs associated with Psychiatric illness (TiC-P) [43]. Total costs will be estimated using a bottom-up (or micro-costing) approach, where units of healthcare usage are multiplied by their appropriate unit cost price and summed to provide an overall total cost estimate [44]. Unit cost prices will be obtained from the latest Dutch guideline for health economic evaluation. Costs of medication (and the pharmacist’s dispensing costs) will be calculated using prices based on Daily Defined Dosage (DDD) taken from www.farmacotherapeutischkompas.nl and www.medicijnkosten.nl. Productivity losses will be based on the friction cost method as per the Dutch guideline. Costs of transport will be calculated as the mean distance per destination multiplied by standard cost prices. If needed, cost prices will be indexed to 2021 using the consumer price index from Statistics Netherlands.

#### Effects

For the CEA, the central clinical end-term will be remission defined as a HDRS-17 depressive symptom severity of less than 8 at 6 months follow-up. For the CUA, the Dutch tariffs of the EQ-5D-5L will be used for computing QALYs [45]. Cumulative costs and QALY gains over the study’s follow-up period will be computed with the area under the curve method. A second CEA will be performed based on the subgroup of patients for which extended follow-up data up until 12 months is available.

#### Combining costs and effects

The comparability of groups at baseline will be assessed for both costs and outcomes, and covariates will be used for adjustment if needed. Missing cost and outcome data will be imputed using multiple imputation with chained equations (MICE) for intention-to-treat (ITT) analysis. Since the trial’s follow-up does not exceed 1 year, no discounting will be performed. The incremental cost-effectiveness ratio (ICER) will be computed to obtain the incremental costs per remitter and the incremental costs per QALY gained. Stochastic uncertainty will be handled using 5000 non-parametric bootstraps and by plotting simulated ICERs on the ICER plane. For decision-making purposes, the ICER acceptability curve will be plotted for various willingness-to-pay (WTP) ceilings for making judgments whether the rTMS added to CBT and antidepressants offers good value for money relative to CBT and switching of antidepressants alone. One-way sensitivity analyses directed at uncertainty in the main cost drivers and outcomes will be performed to assess the robustness of our findings (e.g. under different imputation strategies, e.g. multiple imputation using estimation-maximalization (MIEM)). Both the analysis and reporting of the findings will adhere to the (extended) CONSORT and CHEERS statements [46–48].

## Discussion

The aim of the present study is to examine the (cost-)effectiveness of rTMS combined with CBT compared to pharmacological treatment as usual combined with CBT for the treatment of moderate treatment-resistant unipolar depression without psychotic symptoms. We will also investigate long-term effects up to 6 months and effect decay or preservation up to 12 months. Furthermore, we will explore the effects of both treatments on specific symptoms associated with depression, as well as the effect of expectations regarding the treatment on its effectiveness.

### Strengths and limitations

This study has several strengths. Firstly, since this RCT takes place in a naturalistic clinical care setting, translation of our results to clinical practice will be easier. Regardless of the outcome of this trial, the results can inform clinical decision making and aid in choosing the right treatment for a patient. Another strength lies in the duration of the follow-up, which is longer than previous rTMS trials. Since information up until a year after start of the treatment becomes available, we can also say something about the durability of treatment effects on the longer term and the cost-effectiveness of the intervention over a longer course.

Despite these strengths, some limitations should be considered. First, patients receive medication and CBT as usual care in accordance with the Dutch guidelines. While this reflects clinical practice, treatment heterogeneity is expected, as different psychological and/or pharmacological interventions are provided in different dosages and in varying formats across the participating institutes. Nevertheless, due to the randomized design, the heterogeneity should be similar across conditions.

Another limitation may be that rTMS might lead to improvements in depressive symptoms due to non-specific therapy effects such as more attention by a clinician and a more active lifestyle due to the large number of hospital appointments. A solution would be to use a sham condition, with which these non-specific therapy effects can be assessed, however, the effects of sham protocols have been researched extensively and show that these effects are minimal [49, 50]. As this is a pragmatic trial that aims to assess the comparative efficacy of rTMS and current antidepressant treatments a sham condition would have no added value. Furthermore, non-specific therapy effects can also be expected in the medication group, as these patients will also have more appointments than usual due to the research assessments.

Thirdly, because patients ultimately decide whether they want to participate, there might be a selection bias. Treatment with rTMS especially is time-consuming and intensive, potentially resulting in a selection of highly motivated patients. Importantly, even if rTMS attracts a certain patient population, it can be an effective treatment for depression, not limiting the additional value of this study in examining a (cost-)effective treatment alternative for depression.

Fourth, the CBT in this study differs from earlier studies combining rTMS and CBT, where psychotherapy is offered on an individual basis and during every rTMS session [18]. This design choice was made due to practical reasons, as individual CBT with the same schedule as rTMS is not possible in most hospital settings. Therefore, this could yield slightly different outcomes.

Finally, the follow-up length differs between patients. Whereas we aim to collect follow-up data up until 6 months for all patients, only 70% of patients will be part of the extended follow-up until 12 months. Nevertheless, this is still a large cohort, enabling us to make inferences about the effects of both treatments on the long term. Additionally, even with a smaller sample this information is highly relevant for applicability in clinical practice.

### Clinical implications

With our results, we aim to inform clinical decision making. Currently, rTMS does not have a clear position in the treatment algorithm of guidelines for depression. Though many clinical trials have established rTMS as a safe and effective treatment option for depression, head-to-head comparisons are scarce. Despite its potential as a mainstream treatment for depression, the placement of rTMS in the treatment algorithm is limited by the lack of comparative results, in particular taking its value in combination with CBT into account. The results of this trial will inform us whether rTMS should be considered as treatment alternative to antidepressant medication in patients who have not responded to two previous treatment steps and will give important directions about the proper place of rTMS in treatment guidelines.

## Data Availability

The datasets that will be used and/or analysed during the current study will be made available in the Radboud Repository, https://repository.ubn.ru.nl/.

## Abbreviations

ANCOVA: Analysis of Covariance
ATHF: Antidepressant treatment history form
AUC: Area under the curve
BDI: Beck Depression Inventory
CBT: Cognitive behavioral therapy
CHEERS: Consolidated Health Economic Evaluation Reporting Standards
CEA: Cost-effectiveness analysis
CEQ: Credibility/Expectancy Questionnaire
CONSORT: Consolidated Standards of Reporting Trials
CUA: Cost-utility analysis
DDD: Daily Defined Dosage
DLPFC: Dorsolateral prefrontal cortex
DM-TRD: Dutch Measure for quantification of Treatment Resistance in Depression
DSM: Diagnostic and Statistical Manual of Mental Disorders
DTD: Difficult to treat depression
ECT: Electroconvulsive therapy
EDC: Electronic data capturing
EEG: Electroencephalogram
EQ-5D-5L: EuroQol 5-dimensions 5-levels
ETZ: Elisabeth TweeSteden Ziekenhuis (Elisabeth TweeSteden Hospital)
GGZ: Geestelijke gezondheidszorg (Mental health care)
HDRS: Hamilton Depression Rating Scale
HSDQ: Holland sleep disorders questionnaire
Hz: Hertz
ICER: Incremental cost-effectiveness ratio
iMTA: Institute for Medical Technology Assessment
ITT: Intention to treat
LEIDS-R: Leiden Index of Depression Sensitivity – Revised
MDD: Major depressive disorder
MEP: Motor-evoked potential
MICE: Multiple imputation with chained equations
MIEM: Multiple imputation using estimation-maximalization
MV: MicroVolt
PTQ: Perseverative thinking questionnaire
QALY: Quality-adjusted life year
RCT: Randomized clinical trial
rMT: Resting motor threshold
rTMS: Repetitive transcranial magnetic stimulation
SCID: Structured clinical interview for DSM
SHAPS: Snaith-Hamilton Pleasure Scale
STAI: State/trait anxiety inventory
TCA: Tricyclic antidepressant
TiC-P: Treatment Inventory of Costs in Patients with psychiatric disorders
TRD: Treatment-resistant depression
U.S.A.: United States of America
WTP: Willingness-to-pay
YLD: Years lived with disability
